# Acute exercise and brain BACE1 protein content: a time course study

**DOI:** 10.14814/phy2.14084

**Published:** 2019-04-29

**Authors:** Alex J. Yang, Grant C. Hayward, Rebecca E. K. MacPherson

**Affiliations:** ^1^ Department of Health Sciences Brock University St. Catharines Ontario Canada

**Keywords:** AMPK, beta‐secretase, brain, exercise recovery, obesity

## Abstract

Obesity and insulin resistance are risk factors in the development of neurodegenerative disorders. Previous work suggests that one acute bout of exercise may have beneficial neuro‐protective effects in obese mice. The rate limiting enzyme in the production of amyloid‐beta peptides, BACE1, was reduced in the prefrontal cortex 2 h post‐exercise, however if these effects remain over time is unknown. We aimed to determine how long exercise–induced alterations persist in the prefrontal cortex and hippocampus following a single exercise bout. Male C57BL/6J mice were fed either a low (LFD, 10% kcals from lard) or a high fat diet (HFD, 60% kcals from lard) for 7 weeks. HFD mice then underwent an acute bout of treadmill running (15 m/min, 5% incline, 120 min) followed by 2‐, 8‐, or 24‐h of recovery. The HFD increased body mass (LFD 27.8 ± 1.05 vs. HFD 41.7 ± 0.60 g; *P* < 0.05) and glucose intolerance (AUC LFD 63.27 ± 4.5 vs. HFD 128.9 ± 4.6; *P* < 0.05). Prefrontal cortex BACE1 content was reduced 2‐ and 8‐h post‐exercise compared to sedentary HFD mice, however BACE1 protein content at 24 h was not different. Hippocampal BACE1 content was reduced 8‐ and 24‐h post‐exercise. Compared to the LFD, the HFD had higher prefrontal cortex phosphorylation of p38, JNK, and AMPK, indicative of increased neuronal stress. Post–exercise prefrontal cortex p38 and JNK phosphorylation were no different between the HFD or LFD groups, while ERK phosphorylation was significantly reduced by 24 h. The HFD increased JNK phosphorylation in the hippocampus. These results demonstrate the direct and potent effects of exercise on reducing BACE1 prefrontal cortex and hippocampal content. However the reduction in prefrontal cortex BACE1 content is short lived.

## Introduction

Midlife obesity and insulin resistance are known risk factors for later‐life development of dementia and Alzheimer's disease (AD) (Beydoun et al. 2008; Profenno et al. 2010). This highlights the importance of developing strategies aimed at reducing the risk of AD by targeting obesity and insulin resistance. Exercise training is well known to improve body composition, glucose and insulin tolerance/sensitivity, as well as improve markers of neurodegeneration (Colcombe et al. 2004; Maesako et al. 2012a,b), however the exact cellular and molecular mechanisms underlying the beneficial effects of exercise on neurodegenerative disease remain elusive. Furthermore, it is unknown how much or how frequently one might need to exercise in order to reap neuro‐protective benefits. Our previous work has demonstrated that one bout of exercise can rescue high fat diet (HFD) induced increases in markers of neuroinflammation, cellular stress, and importantly can reduce the protein content of beta‐site amyloid precursor protein cleaving enzyme 1 (BACE1), also known as beta‐secretase (MacPherson et al. 2015a). This research represents the initial steps in systematically establishing prescription–based exercise recommendations to prevent AD and related pathologies, however these results were only measured 2 h post‐exercise and how long the effects of one acute bout of exercise persists is unknown.

Amyloid‐beta plaques are a key feature of AD and consist of extracellular masses of aggregated amyloid‐beta peptides (Orre et al. 2013; Pitt et al. 2013). Amyloid‐beta peptides are detrimental to neuronal networks (Gouras et al. 2005) and play a central role in the molecular mechanisms of early disease progression (Selkoe 2004; Gandy 2005). BACE1 is the rate limiting enzyme involved in the production of amyloid beta peptides through cleavage of the membrane bound amyloid precursor protein (APP). BACE1 is therefore considered a biomarker for early detection, prediction, and progression of AD (Hardy and Higgins 1992; Hampel and Shen 2009).

The exact mechanisms leading to changes in BACE1 protein content are multifaceted, however increased cellular stress and impairments in energy metabolism represent early abnormalities that precede or accompany the initial stages of cognitive impairment (Sims 1996; Steen et al. 2005). For example, AD brains display over active 5'AMP activated protein kinase (AMPK) (Vingtdeux et al. 2011; Ma et al. 2014), as well as increased mitogen activated protein kinase (MAPK) signaling (Hensley et al. 1999; Shoji et al. 2000; Savage et al. 2002). Animal models of obesity and type 2 diabetes provide direct evidence that obesogenic diets accelerate these AD–like pathophysiological changes in the brain. Several studies have demonstrated that high fat feeding of wild‐type mice as well as mouse models of AD (APP transgenic mice) are directly associated with increased amyloid‐beta plaques and altered brain metabolic signaling (Maesako et al. 2012b; Bhat and Thirumangalakudi 2013; MacPherson et al. 2015a; Baranowski and MacPherson 2018).

Exercise training has beneficial effects on whole body energy metabolism and may represent an attractive therapy to reduce or reverse the HFD–induced metabolic disturbances associated with neurodegeneration. Further, exercise training is known to enhance brain function (Colcombe et al. 2004; Kokkinos and Myers 2010) and previous work has demonstrated that voluntary exercise training ameliorates HFD–induced memory deficits and amyloid‐beta deposition in APP transgenic mice (Maesako et al. 2012a,b). However, an important item to note is that exercise training is typically paired with reductions in adiposity and body mass as well as improvements in glucose homeostasis, therefore it is difficult to determine what the direct effects of exercise are on the brain. This point highlights the importance of examining the effects of one acute bout of exercise on the brain. Our previous work has demonstrated the novel effects of one bout of exercise on reducing BACE1 content and reversing HFD–induced markers of inflammation in the prefrontal cortex of obese male mice, independent of weight change (MacPherson et al. 2015a). This work demonstrated that one bout of acute exercise reduces BACE1 protein content and activity and that this was accompanied by a decline in AMPK and MAPK phosphorylation, however, these effects were only observed 2 h post‐exercise. It is possible that the acute exercise bout produced longer‐lasting effects in either the prefrontal cortex or the hippocampus. To increase the utility and efficacy of “exercise as medicine” to prevent or slow the progression of neurodegenerative diseases, it is important to determine how long the beneficial effects of exercise persist and if there are region specific differences in the response to exercise.

The purpose of this study was to examine the time course of the exercise–induced reduction in BACE1 protein content in diet–induced obese male mice. We further aimed to elucidate region specific differences between the prefrontal cortex and the hippocampus. To address this question, we fed C57BL/6 male mice a HFD for 7 weeks, and then examined the effects of an acute bout of exercise on BACE1 protein content and markers of neuroinflammation and stress for 24 h post‐exercise.

## Materials and Methods

### Materials

Molecular weight marker, reagents and nitrocellulose membranes for SDS‐PAGE were purchased from Bio‐Rad (Mississauga, ON). SignalFirePlus ECL reagent (catalogue #12630), protease/phosphatase inhibitor cocktail (catalogue #5872), and SDS loading buffer/dithiothreitol (catalogue #7722) were from Cell Signaling Technologies (Danvers, MA). Horseradish peroxidase–conjugated donkey anti‐rabbit and goat anti‐mouse IgG secondary antibodies were from Jackson ImmunoResearch Laboratories (West Grove, PA). Antibodies against BACE (#5606), ERK (#4695), phospho ERK (threonine 202/tyrosine 204; #9101), p38 (#9212), phospho p38 (threonine 180/tyrosine 182; #9211), JNK (#9252), phospho JNK (threonine 183/tyrosine 185; #4671), GAPDH and VINCULIN were purchased from Cell Signaling (Danvers, MA).

### Animals and diet

Male C57BL/6N mice (8 weeks of age; Charles River) were fed a low (LFD, 10% kcals from lard; Research Diets D12450J; *n* = 9) or a high fat diet (HFD, 60% kcals from lard; Research Diets D12492; *n* = 18) ad libitum for 7 weeks. We have previously demonstrated that this duration and composition of HFD results in obesity and glucose intolerance (MacPherson et al. 2015b). Male mice were utilized to be consistent with our previous work in this area (MacPherson et al. 2015b). Animals were housed individually, had free access to water, and were maintained on a 12/12 h light/dark cycle. All protocols met the guidelines of the University of Guelph Animal Care Committee and the Canadian Council on Animal Care (CCAC).

### Glucose tolerance

During the last week of feeding (week 7) intraperitoneal glucose tolerance tests were performed on 6 h fasted, non‐anesthetized mice from LFD (*n* = 10) and a subset of the HFD mice (*n* = 18). Glucose measures were obtained from tail vein blood using an automated glucometer at baseline and at 15, 30, 45, 60, 90, and 120 min following an intraperitoneal injection of glucose (2 g/kg body mass).

### Acute exercise protocol

To minimize stress differences between groups, both LFD and HFD mice were acclimated to motorized treadmill running during a 3‐day period consisting of 15 min of running/day at 15 m/min, 5% grade. This acclimatization period took place during the last week of feeding. The HFD mice were then assigned into one of two groups, sedentary HFD (HFD *n* = 10) and exercised. Seventy‐four hours following the last day of acclimation, mice ran for 120 min at 15 m/min, with an incline of 5%. The acute bout of exercise started at ~10:00 am, which is the beginning of the light cycle. In the previous work from our lab (MacPherson et al. 2015b), we have found that this duration and intensity (~65% VO_2_max) of exercise is well tolerated. All mice in the exercise treatment group completed the 2 h of treadmill running without issue. Following the exercise bout, mice were placed back in their cages to recover for 2, 8, or 24 h (*n* = 9 per group at each time point).

### Tissue collection

Mice were anesthetized with a weight–adjusted bolus injection of sodium pentobarbital (5 mg/100 g body weight). Thoracic blood was collected for the determination of circulating BDNF. The brains were quickly dissected and left and right prefrontal cortex and hippocampal tissue was collected, snap frozen in liquid nitrogen, and stored at −80 until further analysis (MacPherson et al. 2015a).

### Western blotting

Samples were homogenized (FastPrep®, MP Biomedicals, Santa Ana, CA) in 20 volumes of RIPA lysis buffer (abcam, ab15603) supplemented with protease (Sigma‐Aldrich, 11836170001) and phosphatase inhibitors (Sigma‐Aldrich, 04906845001). The homogenized samples were placed on a shaker in a 4°C fridge for 20 min to reduce foam accumulation. Homogenized samples were then centrifuged at 4°C (15 min @ 10,000*g*), after which the supernatant was collected and protein concentration was determined using a Bicinchoninic acid assay (Sigma‐Aldrich ‐ B9643, VWR – BDH9312). Samples were prepared in loading buffer and then 10–20 *μ*g of protein, depending on the protein, was separated on 10% SDS‐PAGE gels. Protein was wet transferred onto nitrocellulose membranes at 100 V/transfer unit for 1 h. Membranes were blocked in tris buffered saline/0.1% tween 20 (TBST) prepared with 5% nonfat dry milk for 1 h followed by overnight incubation at 4°C with the appropriate primary antibody. Following primary incubation, membranes were rinsed with TBST and incubated with the appropriate Horseradish peroxidase‐conjugated secondary antibodies for 1 h at room temperature. Phosphorylated and total blots were run on duplicate gels. Vinculin and GAPDH were used as loading controls and ponceau staining was also used to confirm equal protein loading. Signals were detected using enhanced chemiluminesence and were subsequently quantified by densitometry using a FluorChem HD imaging system (Alpha Innotech, Santa Clara, CA).

### Statistics

Comparisons between the LFD and HFD groups were made using unpaired, two‐tailed *T*‐tests. A two‐way repeated measures ANOVA (diet and time), followed by a Tukey's post hoc analysis was used to examine differences in LFD and HFD glucose and insulin tolerance tests. Differences in protein content and BDNF over time were determined using a one‐way ANOVA followed by Tukey's post hoc analysis. In cases where data were not normally distributed, logarithmic transformation was used. Data are expressed as means ± SD with significance set at *P* < 0.05.

## Results

### High fat feeding–induced glucose intolerance and increased body mass

**Figure 1:**
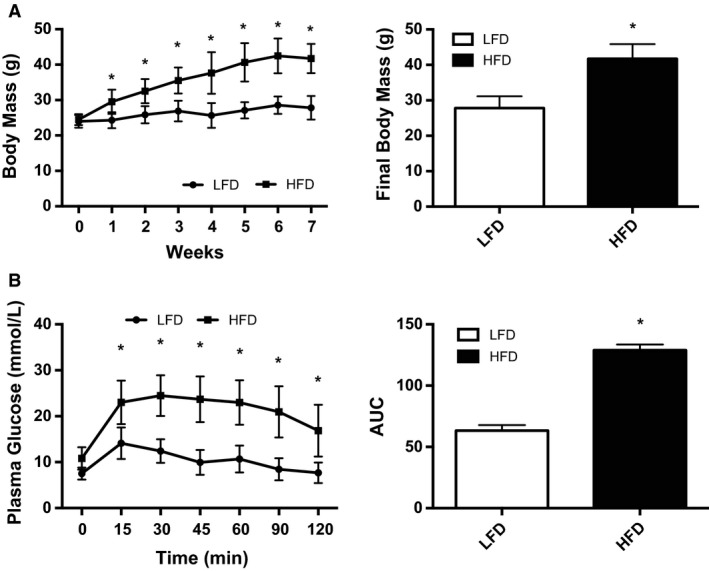
Characterization of diet–induced obesity and glucose intolerance model. (A) Weekly and terminal body mass. (B) Intraperitoneal glucose tolerance test (body weight, 2 g/kg), right panel blood glucose area under the curve. Data are presented as means ± SEM of LFD (*n* = 9) and HFD (*n* = 18) (note that the HFD group includes both the sedentary and exercised mice). *P < 0.05 as determined using a two‐way repeated measures ANOVA (diet and time), followed by Tukey's post hoc analysis.

To induce a state of obesity and glucose intolerance mice were fed a HFD for 7 weeks. The HFD resulted in an increased body mass in comparison to LFD mice (Fig. 1A, *P* < 0.05). Following an intraperitoneal glucose injection, the HFD mice cleared glucose less effectively and had a higher glucose area under the curve (AUC) compared to LFD mice (Fig. 1B, *P* < 0.0001). These results demonstrate that 7 weeks of a HFD resulted in obese, glucose intolerant mice.

### Acute exercise decreases in BACE1 protein content at different time points in the prefrontal cortex and the hippocampus in obese male mice

**Figure 2:**
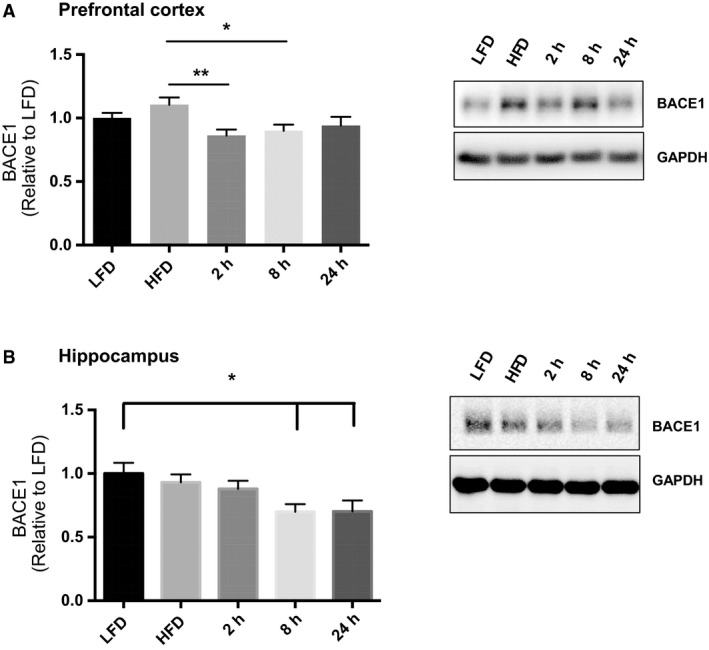
Effect of a HFD and acute exercise on BACE1 content. (A) Acute exercise reduced prefrontal cortex BACE1 protein content 2 h and 8 h post‐exercise. Representative blots are shown beside the quantified data. (B) Acute exercise reduced hippocampal BACE1 protein content 8 and 24 h post‐exercise compared to LFD. Representative blots are shown beside the quantified data. (LFD, *n* = 9; HFD, *n* = 9; 2 h, *n* = 9, 8 h, *n* = 9, 24 h, *n* = 9). Data are presented as means ± SEM. *P < 0.05 as determined using a one‐way ANOVA followed by Tukey's post hoc analysis.

To determine the time course of exercise–induced reductions in BACE1 content we examined BACE1 protein content at 2, 8, and 24 h post‐exercise. In the prefrontal cortex, exercise lowered BACE1 protein content 2 and 8 h post‐ compared to the HFD group (Fig. 2A, ANOVA, *P* = 0.0094; HFD vs. 2 h *P* = 0.0087, HFD vs. 8 h *P* = 0.0352). 24 h post‐exercise BACE1 content was not different from the HFD group, indicating that reductions in BACE1 content in the prefrontal cortex are transient. In the hippocampus, exercise reduced in BACE1 protein content 8 and 24‐h post‐exercise compared to the LFD (Fig. 2B, ANOVA, *P* = 0.0129; LFD vs. 8 h *P* = 0.0381, LFD vs. 24 h *P* = 0.0400).

### Acute exercise rescues HFD induced increases in Akt, AMPK, and MAPK phosphorylation

**Figure 3:**
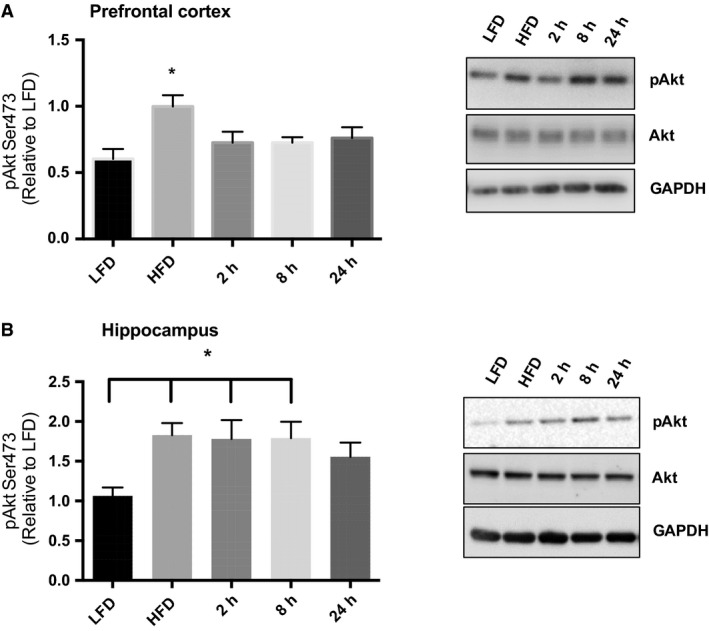
Acute exercise recovers diet–induced MAPK phosphorylation. (A) In the prefrontal cortex the HFD resulted in increased JNK and p38 phosphorylation and this was reduced with an acute bout of exercise. ERK phosphorylation was significantly lower than HFD at 24 h post‐exercise. Representative blots are shown above the quantified data. (B) In the hippocampus the HFD resulted in increased JNK phosphorylation and this was reduced with an acute bout of exercise. (LFD, *n* = 9; HFD, *n* = 9; 2 h, *n* = 9, 8 h, *n* = 9, 24 h, *n* = 9). Data are presented as means ± SEM. *P < 0.05 as determined using a one‐way ANOVA followed by Tukey's post hoc analysis.

Insulin signaling through Akt, as well as AMPK and MAPK signaling are implicated in the up‐regulation of BACE1 content (Savage et al. 2002; Chen et al. 2009), and we previously demonstrated a reduction in Akt, AMPK, and MAPK phosphorylation 2 h post‐exercise (MacPherson et al. 2015a). Therefore, we aimed to examine if these changes remained for 24 h post‐exercise. The high fat diet resulted in an increased in Akt Ser473 phosphorylation in both the prefrontal cortex and the hippocampus. This increased Akt phosphorylation was reduced 2 h post‐exercise in the prefrontal cortex and was maintained for the full 24 h (Fig. 3A; ANOVA: *P* = 0.0125; LFD vs. HFD *P* = 0.0057), while in the hippocampus a reduction in Akt phosphorylation was not observed until 24 h post‐exercise (Fig. 3B; ANOVA *P* = 0.0199; LFD vs. HFD *P* = 0.034, LFD vs. 2 h *P* = 0.054, LFD vs. 8 h *P* = 0.04).

**Figure 4:**
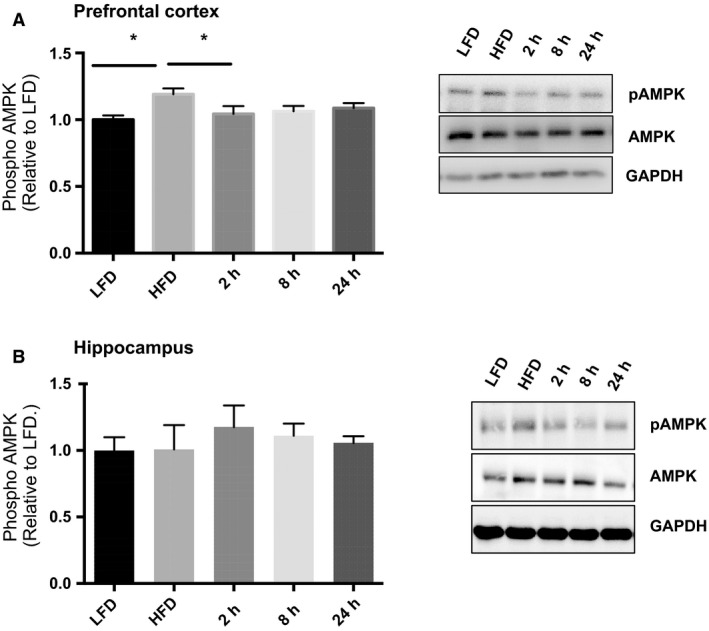
Acute exercise recovers diet–induced aberrant Akt signaling. (A) In the prefrontal cortex the HFD resulted in increased Akt Ser473 phosphorylation and this was reduced with one bout of acute exercise. (B) In the hippocampus the HFD resulted in increased Akt Ser473 phosphorylation and this was reduced 24 h postacute exercise. (LFD, *n* = 9; HFD, *n* = 9; 2 h, *n* = 9, 8 h, *n* = 9, 24 h, *n* = 9). Data are presented as means ± SEM. *P < 0.05 as determined using a one‐way ANOVA followed by Tukey's post hoc analysis.

AMPK threonine 172 phosphorylation was significantly increased in the prefrontal cortex of the HFD sedentary group (Fig. 4A; ANOVA: *P* = 0.0343; LFD vs. HFD *P* = 0.0022) with reduced AMPK phosphorylation compared to HFD at the 2 h time point (HFD vs. 2 h *P* = 0.0196). There were no significant changes in AMPK phosphorylation in the hippocampus with the HFD or the acute bout of exercise (Fig. 4B; ANOVA *P* = 0.8554).

**Figure 5:**
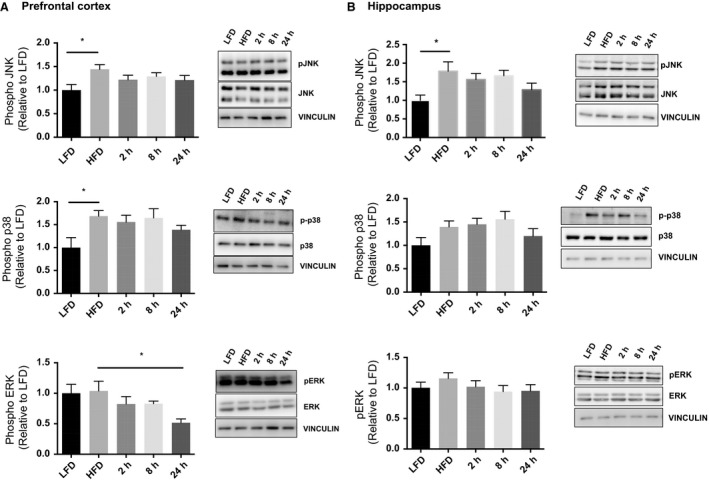
Acute exercise decreases AMPK phosphorylation. HFD increased AMPK phosphorylation compared to LFD. (A) Representative blots are shown beside the quantified data. (B) No changes were observed in hippocampal AMPK phosphorylation with diet or acute exercise. (LFD, *n* = 9; HFD, *n* = 9; 2 h, *n* = 9, 8 h, *n* = 9, 24 h, *n* = 9). Data are presented as means ± SEM. *P < 0.05 as determined using a one‐way ANOVA followed by Tukey's post hoc analysis.

Phosphorylation of JNK and p38 was increased in the prefrontal cortex following the HFD and the acute bout of exercise resulted in no difference over the 24 h recovery period when compared to LFD and HFD groups (Fig. 5A; JNK ANOVA: *P* = 0.0440, LFD vs. HFD *P* = 0.0237; p38 ANOVA: *P* = 0.0324; LFD vs. HFD *P* = 0.0209). Phosphorylation of ERK was not different between LFD and HFD groups in the prefrontal cortex and was decreased 24 h post‐exercise (Fig. 5A, ANOVA *P* = 0.0270; HFD vs. 24 h *P* = 0.0358). HFD also increased JNK phosphorylation in the hippocampus (Fig. 5B; ANOVA *P* = 0.0339; LFD vs HFD *P* = 0.036), with no changes observed in either phosphorylated p38 or ERK (*P* > 0.05).

## Discussion

This study examined the direct effect of exercise on BACE1 content in the prefrontal cortex and the hippocampus for 24 h post‐exercise. Our novel results demonstrate that prefrontal cortex BACE1 protein content is lower 2‐ and 8‐h post‐exercise, however by 24 h post‐exercise the reduction in BACE1 protein content is no longer significant. While, hippocampal BACE1 protein content is reduced at 8‐ and 24‐h post‐exercise. Together, this not only highlights the potent effects of exercise on the brain but also highlights regional differences in the direct response to exercise. These changes in BACE1 content were accompanied by a reduction in diet–induced markers of aberrant insulin signaling (phosphorylated Akt), neuroinflammation and stress (phosphorylated AMPK, JNK, and p38) in the prefrontal cortex and a recovery of Akt and ERK phosphorylation in the hippocampus over the 24 h post‐exercise period. Together, our results suggest that a single, acute bout of exercise has direct beneficial neuro‐protective effects in obesity.

Diet–induced obesity and glucose intolerance are associated with AD–like pathological changes in the brain. BACE1 is the rate limiting enzyme involved in the production of amyloid‐beta peptides. We have previously demonstrated that prefrontal cortex BACE1 protein content is reduced in diet–induced obese male mice 2‐h after one bout of treadmill exercise. Here we examined the time course of this reduction in protein content over a 24‐h period post‐exercise. Our novel results demonstrate that prefrontal cortex BACE1 protein content is lower 2‐ and 8‐h post‐exercise, however by 24 h post‐exercise the reduction in BACE1 protein content is no longer significant. Importantly, this indicates that the exercise–induced reduction in BACE1 is transient in the prefrontal cortex and that to accomplish long‐term reductions in BACE1, repeated exercise bouts are likely needed. Interestingly, in the hippocampus, BACE1 protein content was reduced at 8‐ and 24‐h post‐exercise demonstrating that one bout of exercise can have a longer‐lasting effect on BACE1 content in the hippocampal region. This finding is important and demonstrates that exercise can have a direct effect on BACE1 content.

Current therapies for patients with AD have a limited efficacy in improving symptoms, and they do not treat the underlying cause of the disease. Due to the detrimental role of amyloid‐beta production, reducing BACE1 is an attractive approach in treating Alzheimer's disease (Cheng et al. 2015; May et al. 2015; Kennedy et al. 2016; Mullard 2017; Thaisrivongs et al. 2018; Ye et al. 2018). There have been several clinical trials with BACE1 inhibitors that have shown to be effective at decreasing amyloid plaque levels, however many of them do not improve cognition and have been discontinued due to undesired effects (Vassar 2014; Yan 2016; Mullard 2017; Thaisrivongs et al. 2018; Volloch and Rits 2018; Ye et al. 2018). Together, this highlights the importance of identifying novel treatments that act on the underlying pathophysiology of AD and have the potential to modulate disease progression. Our current work demonstrates that one bout of moderate intensity endurance type exercise can reduce the protein content of BACE1. This finding is important as it indicates that daily physical activity may be necessary to result in prolonged or chronic reductions in BACE1.

In addition to the exercise–induced changes in BACE1 protein content, our results demonstrate that one exercise bout reduces the increase in diet–induced markers of neuroinflammation and stress. Specifically, in the prefrontal cortex 2 h post‐exercise phosphorylated Akt, AMPK, JNK, and ERK were returned to levels similar to the LFD control group. Additionally, 24 h post‐exercise phosphorylated ERK was still significantly reduced compared to the HFD sedentary group. Work examining brain samples from patients with AD has demonstrated that neurons and dystrophic neurites have higher JNK (Shoji et al. 2000; Zhu et al. 2001b), p38 (Hensley et al. 1999; Zhu et al. 2000), and ERK activation (Ferrer et al. 2001). The simultaneous activation of ERK and JNK is thought to represent one of the earliest events in the disease pathogenesis that precipitates further alterations (Zhu et al. 2001a). It is thought that increases in AMPK activation in the brain are indicative of energetic stress or perturbed brain metabolism. Brain samples from patients with AD demonstrate abnormally activated AMPK (Vingtdeux et al. 2011; Ma et al. 2014) and previous work has determined that increased AMPK activity can lead to increased BACE1 protein content (Chen et al. 2009). Our results show that high fat feeding resulted in a significant increase in phosphorylated AMPK and that acute aerobic exercise resulted in a reduction in phosphorylated AMPK after 2 h of recovery. This result demonstrates the effectiveness of exercise in reducing energetic stress and restoring metabolic balance in the brain.

Here we have demonstrated the potency of one exercise bout in reversing HFD–induced alterations in the brain in male mice. These results are promising and support exercise as a potential preventative medicine for the development of neurodegenerative diseases, such as AD. However, if the same effects remain in female mice is unknown and future work should examine sex‐related differences in response to exercise. Further, the exact neurobiological bases underlying the benefits of physical exercise remain to be elucidated, however a number of studies suggest that the synthesis and release of trophic factors, particularly brain‐derived neurotrophic factor (BDNF), play a crucial role mediating the neuro‐protective effects of exercise on the brain (MacPherson 2017). Future studies should aim to examine a direct link between BDNF and the reduction in BACE1 content.

Together, the findings from the current study highlight the therapeutic potential of exercise, independent of alterations in body mass or adiposity, as a tool to ameliorate changes in early AD–associated pathology. We present novel insight into the duration of these beneficial effects over a 24 h period. This research represents the initial steps in systematically establishing prescription–based exercise recommendations to prevent AD and related pathologies. The results of this research will provide clinically relevant information and can guide health care practitioners in providing meaningful and precise advice to at risk populations about the optimal frequency of exercise needed to prevent brain dysfunction and cognitive decline. Our results highlight the importance of daily exercise interventions for long‐term improvements in early AD–associated pathology.

## Conflict of Interest

There are no conflicts of interest to disclose.
